# Supplementary material for: PKCδ is an activator of neuronal mitochondrial metabolism that mediates the spacing effect on memory consolidation
Source: bioRxiv. 2024 Jun 18:2023.10.06.561186. Preprint. [Version 2] doi: 10.1101/2023.10.06.561186 (PMC11212906; doi:10.1101/2023.10.06.561186)
Supplement: Supplement 2 [file NIHPP2023.10.06.561186v2-supplement-2.pdf]

bioRxiv preprint doi: https://doi.org/10.1101/2023.10.06.561186; this version posted June 18, 2024. The copyright holder for this preprint (which was not certified by peer review) is the author/funder, who has granted bioRxiv a license to display the preprint in perpetuity. It is made available under aCC-BY 4.0 International license.

Supplemental information

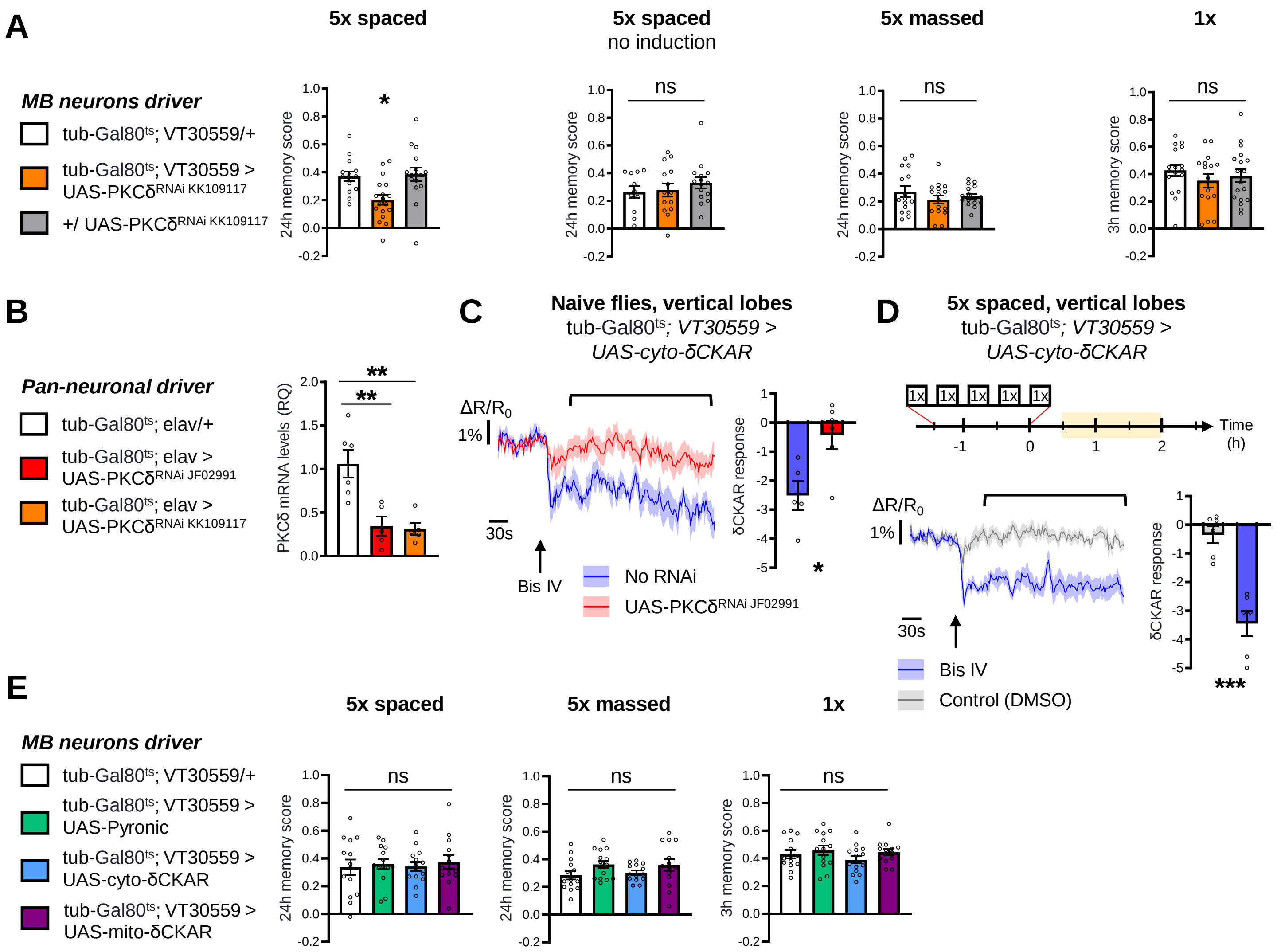

Figure 1 – figure supplement 1. Control experiments for behavior analysis and δCKAR imaging experiments.

(A) Behavior analysis with a second non-overlapping PKCδ RNAi: 24-h memory after 5x spaced conditioning was specifically impaired after induction of the PKCδ RNAi expression in adult MB (n=14-18, $F_{2,45}$=6.76, p=0.027), and it was not impacted without induction (n=11-15, $F_{2,37}$=0.62, p=0.54). No memory defect was found after 5x massed training (n=16, $F_{2,45}$=0.90, p=0.42) or 1x training (n=17-18, $F_{2,48}$=0.66, p=0.52) in flies knocked down for PKCδ in adult MB. **(B)** Pan-neuronal expression of either PKCδ RNAi at the adult stage significantly reduced PKCδ mRNA levels. Relative Quantification (RQ) was performed, indicating the foldchange of mRNA levels relative to the control genotype (n=5-6, $F_{2,13}$=11.85, p=0.0012). **(C)** In naive flies, application of 5 µM of Bis IV (black arrow), a PKC inhibitor, resulted in a decrease in the cyto-δCKAR response, and this response was abolished when PKCδ was knocked down (n=5-

bioRxiv preprint doi: https://doi.org/10.1101/2023.10.06.561186; this version posted June 18, 2024. The copyright holder for this preprint (which was not certified by peer review) is the author/funder, who has granted bioRxiv a license to display the preprint in perpetuity. It is made available under aCC-BY 4.0 International license.

6, t_9=3.01, p=0.015). Quantification of the mean cyto-δCKAR response was performed 20 s after Bis IV application on a time window of 250 s (black line). **(D)** After 5x spaced training, Bis IV injection also decreased the cyto-δCKAR response as compared to the DMSO control (n=6, t_{10}=4.83, p=0.0007). **(E)** Memory formed after either 5x spaced (n=14, $F_{3,52}$=0.14, p=0.94), or 5x massed (n=14, $F_{3,52}$=1.69, p=0.18), or 1x training (n=14, $F_{3,52}$=1.07, p=0.37) was not impaired by the expression in the MB neurons at adult stage of the imaging probes used in this study: Pyronic, cyto-δCKAR and mito-δCKAR. Data are expressed as mean ± SEM with dots as individual values, and were analyzed either by one-way ANOVA with post hoc testing by the Tukey pairwise comparisons test (A-B and E), or by an unpaired two-sided t-test (C-D). Asterisks refer to the least significant P-value of post hoc comparison between the genotype of interest and the genotypic controls (A-B) or to the P-value of the unpaired t-test comparison (C-D) using the following nomenclature: *p<0.05, **p<0.01, ***p<0.001, ns: not significant, p>0.05. Supplementary Table 1.

bioRxiv preprint doi: https://doi.org/10.1101/2023.10.06.561186; this version posted June 18, 2024. The copyright holder for this preprint (which was not certified by peer review) is the author/funder, who has granted bioRxiv a license to display the preprint in perpetuity. It is made available under aCC-BY 4.0 International license.

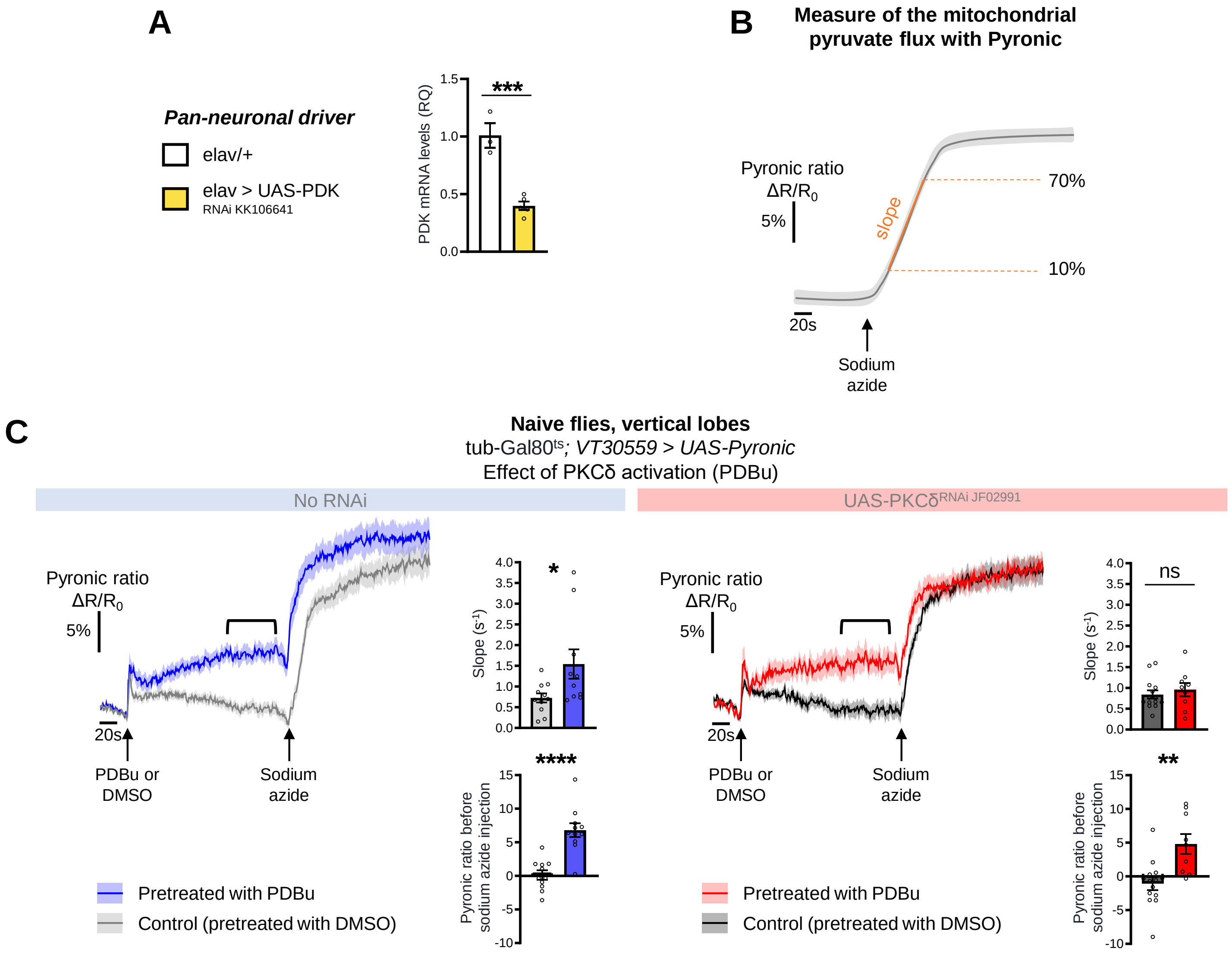

Figure 3 – figure supplement 1. PKCδ activation by PDBu application increases the MB neuronal pyruvate flux.

(A) Pan-neuronal expression of PDK RNAi significantly reduced PDK mRNA levels, as compared to the control genotype (n=3-5, t_6=6.66, p=0.0006). **(B)** Schema representing the measure of the mitochondrial pyruvate flux using the pyruvate FRET sensor Pyronic. Here, mitochondrial respiration is blocked by sodium azide at the beginning of the recording, thereby stopping pyruvate mitochondrial uptake. The expected kinetic of pyruvate accumulation following the azide treatment is represented in grey, showing that pyruvate accumulates from the arrest of its mitochondrial uptake until saturation of the sensor. The rate of pyruvate accumulation (i.e. the slope of the measured kinetic, measured between 10 and 70% of the plateau) reflects the rate at which pyruvate was consumed by mitochondria for energy production before their blockade. **(C)** The Pyronic probe was expressed in adult MB neurons and the pyruvate FRET signal was quantified in the vertical lobes. In naive control flies, PDBu pretreatment (250 µM, 3 min) elicited a faster pyruvate accumulation following sodium azide application (5 mM, black arrows) as compared to flies pretreated with the DMSO solvent alone

bioRxiv preprint doi: https://doi.org/10.1101/2023.10.06.561186; this version posted June 18, 2024. The copyright holder for this preprint (which was not certified by peer review) is the author/funder, who has granted bioRxiv a license to display the preprint in perpetuity. It is made available under aCC-BY 4.0 International license.

(left panel, slope measurement n=10-11, t_{19}=2.31, p=0.032). This PDBu pretreatment induced increase in pyruvate accumulation is abolished when PKCδ is knocked down in MB neurons as compared to DMSO pretreatment (right panel, slope measurement n=9-14, t_{21}=0.67, p=0.51). Notably, in control flies, PDBu injection is followed by a progressive increase in the measured Pyronic ratio (black line) as compared to DMSO injection (n=11, t_{20}=5.44, p<0.0001). This effect is not sensitive to PKCδ knockdown (n=9-14, t_{21}=3.54, p=0.0019). As PDBu is known to activate other PKCs (Wu-zhang et al., 2012), this observation likely reflects a PDBu off-target effect independent of PKCδ on pyruvate neuronal levels. Data are expressed as mean ± SEM with dots as individual values, and were analyzed by unpaired two-sided t-test. Asterisks refer to the P-value of the unpaired t-test comparison using the following nomenclature: *p<0.05, **p<0.01, ***p<0.001, ****p<0.0001, ns: not significant, p>0.05.

bioRxiv preprint doi: https://doi.org/10.1101/2023.10.06.561186; this version posted June 18, 2024. The copyright holder for this preprint (which was not certified by peer review) is the author/funder, who has granted bioRxiv a license to display the preprint in perpetuity. It is made available under aCC-BY 4.0 International license.

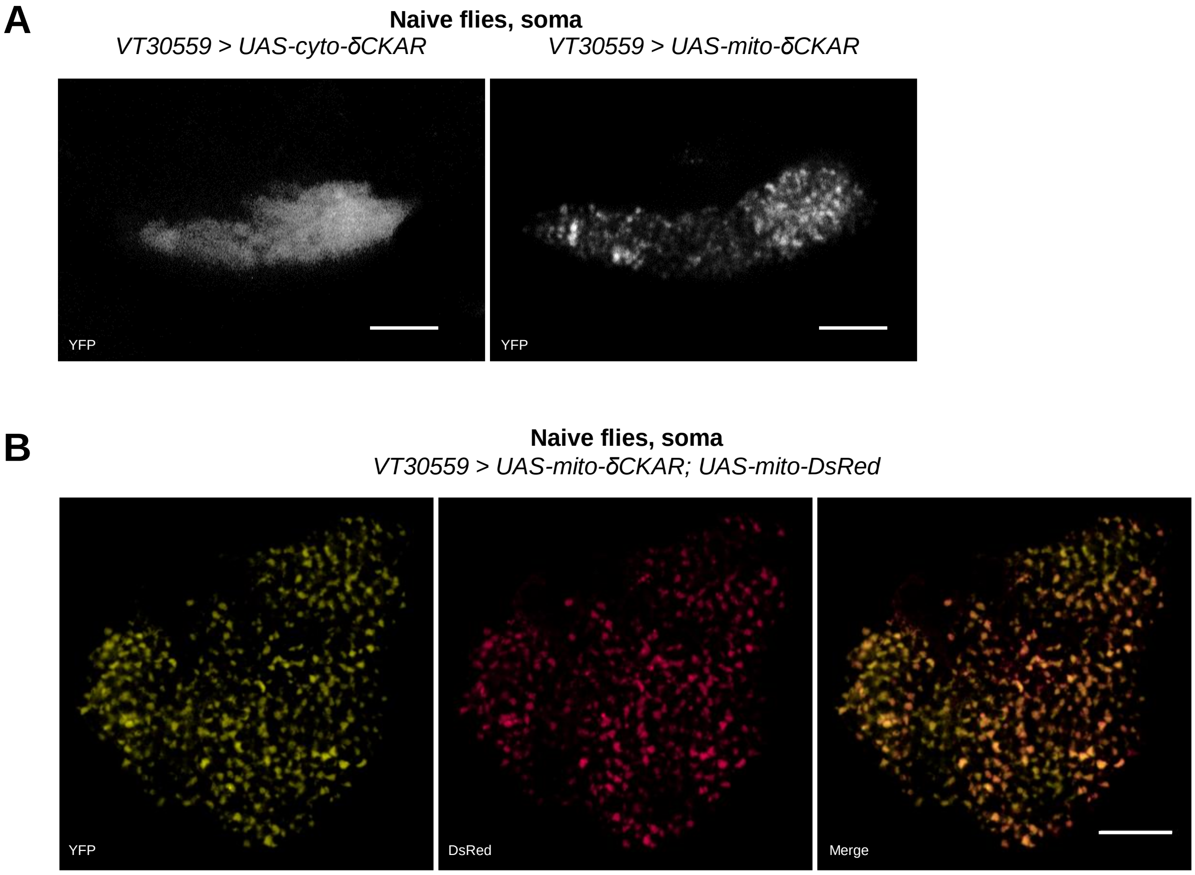

Figure 4 – figure supplement 1. Subcellular addressing of the cyto-δCKAR and mito-δCKAR probes.

(A) Comparison of fluorescence sub localization in the soma of MB neurons in flies expressing either cyto-δCKAR or mito-δCKAR under the control of the VT30559 driver. Mito-δCKAR punctate signal, suggests a mitochondrial localization of the probe, in contrast to the diffuse signal observed with cyto-δCKAR. This comparison was done in the soma area of the MBs because the density of mitochondria in the lobes is too important to allow their discrimination. Images were acquired by 2-photon microscopy and the cyto-and mito-δCKAR sensors were visualized through the YFP channel. **(B)** Coexpression of mito-δCKAR and mito-DsRed (DsRed is addressed to mitochondria using the COX8 targeting sequence (Lutas et al., 2012)) in the MB neurons shows a clear colocalization of the YFP and DsRed signals. The image acquired by confocal microscopy shows a single plan located in the middle of a MB soma of a representative fly. Scale bars=20 µm. See full stack on Video 1.

bioRxiv preprint doi: https://doi.org/10.1101/2023.10.06.561186; this version posted June 18, 2024. The copyright holder for this preprint (which was not certified by peer review) is the author/funder, who has granted bioRxiv a license to display the preprint in perpetuity. It is made available under aCC-BY 4.0 International license.

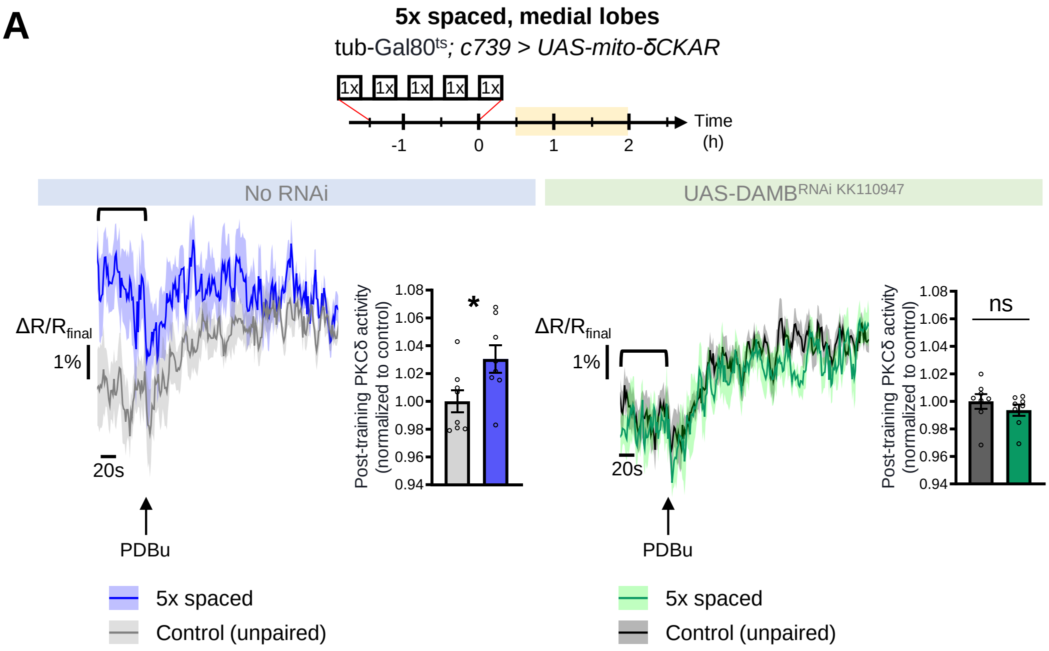

Figure 5 – figure supplement 1. PKCδ also translocates to the mitochondria of the β lobe upon LTM formation.

(A) The post-training activity of mitochondrial PKCδ was measured in the medial lobes between 30 min and 2 h post-conditioning (in yellow on the imaging time frame), in the same flies as in Figure 5A-B. PKCδ activity was increased in the medial lobes after 5x spaced conditioning as compared to unpaired conditioning (left panel n=8, t_{14}=2.38, p=0.032). On the other hand, PKCδ activity was unchanged in the medial lobes after 5x spaced conditioning upon the knock-down of DAMB in adult α/β neurons, as compared to unpaired conditioning (right panel n=8, t_{14}=0.95, p=0.36). Data are expressed as mean ± SEM with dots as individual values, and were analyzed by unpaired two-sided t-test. Asterisks refer to the P-value of the unpaired t-test comparison using the following nomenclature: *p<0.05, ns: not significant, p>0.05.

bioRxiv preprint doi: https://doi.org/10.1101/2023.10.06.561186; this version posted June 18, 2024. The copyright holder for this preprint (which was not certified by peer review) is the author/funder, who has granted bioRxiv a license to display the preprint in perpetuity. It is made available under aCC-BY 4.0 International license.

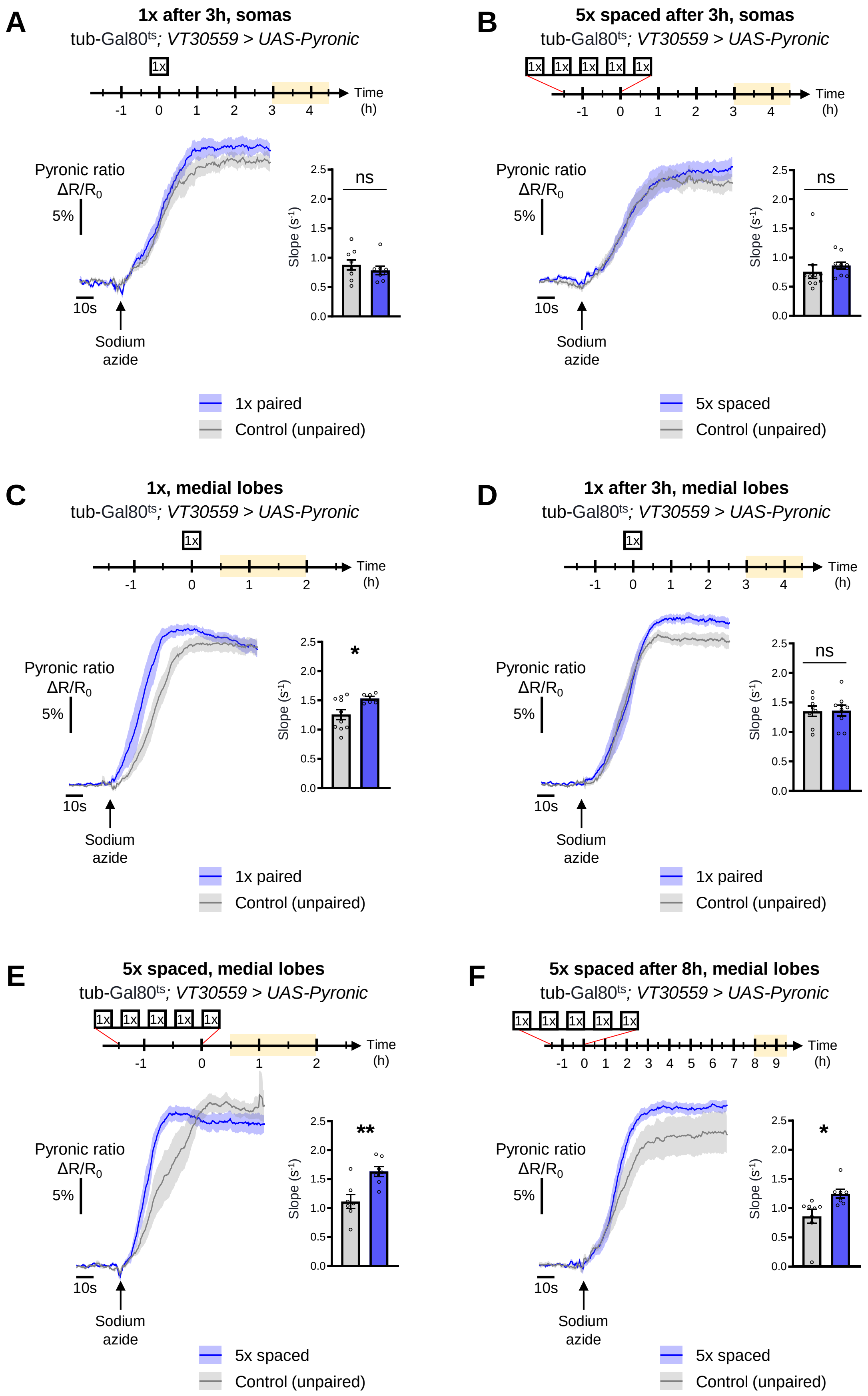

1343 **Figure 6 – figure supplement 1. Additional characterization of the temporal dynamic of**
1344 **the pyruvate flux following conditioning in the somas and medial lobes.**

bioRxiv preprint doi: https://doi.org/10.1101/2023.10.06.561186; this version posted June 18, 2024. The copyright holder for this preprint (which was not certified by peer review) is the author/funder, who has granted bioRxiv a license to display the preprint in perpetuity. It is made available under aCC-BY 4.0 International license.

(A) At the level of the MB somas, 3 h to 4 h 30 min after 1x conditioning, the pyruvate flux of control flies was not increased as compared to 5x spaced unpaired conditioning (slope measurement n=8-9, t_{15}=0.85, p=0.41). **(B)** 3 h to 4 h 30 min after 5x spaced conditioning, the pyruvate flux of control flies was not increased as compared to 5x spaced unpaired conditioning at the level of the MB somas (slope measurement n=10, t_{18}=0.81, p=0.43). **(C)** 30 min to 2h after 1x paired conditioning, an increased pyruvate flux was measured in control flies at the level of the medial lobes as compared to unpaired 1x training (slope measurement n=6-10, t_{14}=2.37, p=0.033). **(D)** 3 h to 4 h 30 min after 1x paired conditioning of control flies the rate of pyruvate accumulation in the medial lobes was similar as compared to unpaired conditioning (slope measurement n=8-9, t_{15}=0.081, p=0.94). **(E)** 30 min to 2 h after 5x spaced conditioning, the pyruvate flux of control flies was increased in the medial lobes as compared to 5x spaced unpaired conditioning (slope measurement n=7, t_{12}=3.46, p=0.0047). **(F)** 8 h to 9 h 30 min after the last cycle of 5x spaced conditioning of control flies, the pyruvate flux was still increased in the medial lobes as compared to 5x spaced unpaired conditioning (slope measurement n=7-8, t_{13}=2.62, p=0.021). Data are expressed as mean ± SEM with dots as individual values, and were analyzed by unpaired two-sided t-test. Asterisks refer to the P-value of the unpaired t-test comparison using the following nomenclature: *p<0.05, **p<0.01, ns: not significant, p>0.05.

bioRxiv preprint doi: https://doi.org/10.1101/2023.10.06.561186; this version posted June 18, 2024. The copyright holder for this preprint (which was not certified by peer review) is the author/funder, who has granted bioRxiv a license to display the preprint in perpetuity. It is made available under aCC-BY 4.0 International license.

Supplementary Table 1. Sensory acuity controls, related to Figure 1 and Figure 2.

Genotypes	Shock Avoidance		Naive odor avoidance			
			Octanol		Methylcyclohexanol	
	Mean ± s.e.m.	Statistics	Mean ± s.e.m.	Statistics	Mean ± s.e.m.	Statistics
tubGal80^{ts};VT30559/+	0.63±0.057	$F_{2,33}$=2.60	0.74±0.053	$F_{2,27}$=5.60	0.72±0.069	$F_{2,27}$=1.27
tubGal80^{ts};VT30559 > UAS-PKCδ$^{RNAi\ JF02991}$	0.76±0.033	p=0.090	0.53±0.070	p=0.0092**	0.60±0.082	p=0.30
UAS-PKCδ$^{RNAi\ JF02991}$/+	0.64±0.039	n=12	0.44±0.073	n=9-11	0.56±0.069	n=9-11
tubGal80^{ts};VT30559/+	0.47±0.052	$F_{2,33}$=0.38	0.63±0.040	$F_{2,33}$=2.76	0.66±0.046	$F_{2,33}$=0.64
tubGal80^{ts};VT30559 > UAS-PKCδ$^{RNAi\ KK109117}$	0.42±0.039	p=0.69	0.70±0.033	p=0.078	0.67±0.034	p=0.53
UAS-PKCδ$^{RNAi\ KK109117}$/+	0.43±0.047	n=12	0.57±0.047	n=12	0.61±0.050	n=12
tubGal80^{ts};c739/+	0.45±0.060	$F_{2,39}$=0.61	0.58±0.050	$F_{2,33}$=2.48	0.46±0.044	$F_{2,33}$=0.94
tubGal80^{ts};c739 > UAS-PKCδ$^{RNAi\ JF02991}$	0.40±0.053	p=0.55	0.45±0.036	p=0.099	0.47±0.051	p=0.40
UAS-PKCδ$^{RNAi\ JF02991}$/+	0.49±0.059	n=14	0.48±0.045	n=12	0.54±0.048	n=12

Control experiments for olfactory acuity and electric shock avoidance: the expression of either of the two PKCδ RNAi used in this study, in MB neurons at the adult stage had no significant effect on olfactory acuity, or on the avoidance of electric shocks.

**: Tukey post hoc comparison between the genotype of interest and controls are not significant:

tubGal80^{ts};VT30559 > UAS-PKCδ$^{RNAi\ JF02991}$ vs tubGal80^{ts};VT30559/+ : ns

tubGal80^{ts};VT30559 > UAS-PKCδ$^{RNAi\ JF02991}$ vs UAS-PKCδ$^{RNAi\ JF02991}$/+ : ns

tubGal80^{ts};VT30559/+ vs UAS-PKCδ$^{RNAi\ JF02991}$/+ : **

Video 1. Video of mito-δCKAR and mito-DsRed colocalization z-stack. Video of the full z-stack composed of 28 images showing the entire MB soma of the representative fly shown on Figure 4 – figure supplement 1B. The YFP channel is on the left, DsRed is in the middle, and the merged channels are shown on the right. Scale bar=20 µm.
